# Dopamine transporter single photon emission computed tomography (DaT-SPECT) use in the diagnosis and clinical management of parkinsonism: an 8-year retrospective study

**DOI:** 10.1007/s00415-023-11563-y

**Published:** 2023-02-16

**Authors:** Kaki Tsang, Richard Walker

**Affiliations:** 1 North Tees and Hartlepool Trust, Stockton-upon-Tees, UK; 2 Department of Population Health Sciences Institute, Newcastle University, Newcastle upon Tyne, UK; 3 Northumbria Healthcare NHS Foundation Trust, Medicine, North Shields, Tyne and Wear, UK

**Keywords:** Parkinsonism, Dopamine transporter scan, Transporter single photon emission computed tomography, Diagnosis, Clinical management

## Abstract

**Introduction:**

Parkinson’s disease (PD) is a neurodegenerative movement disorder that is typically diagnosed clinically. DaT-SPECT scanning (DaT Scan) can be used when there is diagnostic difficulty differentiating from non-neurodegenerative Parkinsonism. This study assessed the effect of DaT Scan imaging on diagnosis and subsequent clinical management of these disorders.

**Methods:**

This single-trust retrospective study involved 455 patients who had undergone DaT scans for investigation for Parkinsonism, between 01/01/2014 and 31/12/2021. Data collected included patient demographics, date of clinical assessment, scan report, pre-scan and post-scan diagnosis, and clinical management.

**Results:**

The mean age at scan was 70.5 years and 57% were male. The percentage of patients who had an abnormal scan result was 40% (*n* = 184), whilst 53% (*n* = 239) had a normal scan result, and 7% (*n* = 32) had an equivocal scan. Pre-scan diagnosis was consistent with scan results in 71% of cases of neurodegenerative Parkinsonism, whereas this figure was 64% for cases of non-neurodegenerative Parkinsonism. For all DaT scans, the diagnosis was changed in 37% of patients (*n* = 168), whilst the clinical management was changed in 42% of patients (*n* = 190). Change in management involved 63% starting dopaminergic medication, 5% stopping dopaminergic medications, and 31% undergoing other changes in management.

**Conclusion:**

DaT imaging is useful for confirming the correct diagnosis and clinical management for patients with clinically indeterminate Parkinsonism. Pre-scan diagnoses were generally consistent with scan results.

## Introduction

Parkinson’s disease (PD) is a syndrome characterised by the loss of dopaminergic neurones due to a neurodegenerative process. It is generally diagnosed clinically, and has been defined by the UK Parkinson’s disease Society Brain Bank Criteria as features of bradykinesia with either tremor, rigidity or postural instability, after other causes have been ruled out [1].

There are, however, often difficulties in differentiating neurodegenerative Parkinson’s disorders from other types of non-degenerative Parkinsonism, e.g., vascular or drug-induced Parkinson’s, or non-Parkinsonian tremor, e.g., essential tremor, in clinical practice [2]. Conditions that fall within the remit of the latter can vary. The accuracy of clinical diagnosis of PD by movement disorder specialists was determined to be 79.6%, with the accuracy increasing up to 82.7% when using set criteria to aid diagnosis [3]. This therefore suggests that using clinical diagnosis alone would result in a considerable proportion of patients being misdiagnosed [3].

Dopamine transporter (DaT) ^123^I-FP-CIT single photon emission computed tomography (SPECT) is an imaging technique used to help distinguish between neurodegenerative Parkinson’s disorders with dopaminergic deficit from other causes of Parkinsonism. DaT-SPECT has been found to have a sensitivity of up to 90%, and specificity of up to 92% in distinguishing between idiopathic PD compared to non-neurodegenerative causes of Parkinsonism [4].

A recent systematic review and meta-analysis of the current literature has described that DaT scans contributed to changing the diagnosis in almost a third of patients, and management was changed in approximately half of patients scanned [5]. However, the review described that there is limited research regarding subgroup analysis when assessing the effect of DaT scans on clinical management [5].

The purpose of this study is to assess the effect of DaT scans on diagnosis and clinical management of suspected Parkinsonian Syndrome from 2014 to 2021 within a movement disorder service. This includes subgroup analysis depending on the clinical indication of the scan, as well as analysis of the duration between receiving scans to receiving treatment to better reflect changes in management due to the DaT scan result in actual practice.

## Methods

This retrospective study was performed in an established Parkinson’s outpatients’ service at Northumbria Healthcare NHS Foundation Trust. All patients who received a DaT scan under the PD service from 01/01/2014 to 31/12/2021 were included in the study. A list of all the DaT scans undertaken on patients cared for by the trust was compiled and the patients who had not received a DaT Scan under the PD service were excluded. This was because DaT scans undertaken by other specialties were often used for the purpose of clinical confirmation of other conditions not pertaining to movement disorders. For example, DaT scans performed under psychiatry to help distinguish between types of psychiatric disorders, with no features of movement disorders, were excluded. Other exclusion criteria included if patients’ clinical notes were insufficient or if the patient was lost to follow-up, so the primary outcomes could not be determined. Any additional information required was identified in the electronic clinical notes system by an independent clinician, who was not involved in the care of the patients seen in the Parkinson’s service (KT).

Data collected included patient demographics, date of clinical assessment, dates of scan and scan reporting, clinical indications (pre-scan diagnosis), scan report results, post-scan diagnosis, and clinical management. Clinical indications were grouped into PD, Parkinson’s Plus Syndromes, Lewy-body dementia (LBD), Drug-Induced Parkinson’s (DIP), Vascular Parkinson’s (VP), Essential tremor (ET), and other conditions. The scan outcomes were categorised into three groups: abnormal (positive scan), normal (negative scan), and equivocal scan.

Conditions included within Parkinson’s Plus Syndromes were progressive supranuclear palsy (PSP), multiple system atrophy, and corticobasal degeneration. Conditions included within other conditions were ventroperitoneal shunt, normal pressure hydrocephalus, cerebral myelomalacia/encephalomalacia, and anxiety-induced tremor. Patients who started treatment, who experienced a change in the dose or type of treatment, or who stopped treatment as a result of the scan result were identified as undergoing changes in clinical management. Patients who stayed on the same treatment or who were not started on treatment were counted as having no change to clinical management.

The decision for DaT Scan referral was made by movement disorder specialists when there was diagnostic uncertainty or when diagnostic confirmation was needed. The DaT Scans were performed by an independent radiology department with no involvement in the patient management. Institutional approval was obtained before the beginning of the data collection.

## Results

From 2014 to 2021, a total of 455 scans were requested by the Parkinson’s team. A slight majority were male (57%), and the average age at the time of scan was 70.5 years (range 36–90 years). Analysis of individual years demonstrated that the average age at which patients were receiving DaT scan has been steadily increasing between 2014 and 2021. In all years, there was a male majority. The median number of days from clinical assessment to having the DaT scan report was 35 days. Table 1 describes basic patient demographics from 2014 to 2021.

**Table 1:** Basic patient demographic from 2014 to 2021

Demographics	2014	2015	2016	2017	2018	2019	2020	2021	Total
Mean age (years)	68.8	66.8	65.3	65.0	72.9	72.4	71.6	74.2	70.5
Male (*n*)	29	30	27	13	26	55	33	46	259
Female (*n*)	22	25	22	8	18	32	25	43	195
Male (%)	56.9	54.5	55.1	61.9	59.1	63.2	55.9	51.7	56.9
Female (%)	43.1	45.5	44.9	38.1	40.9	36.8	44.1	48.3	42.9
Number of scans (*n*)	51	55	49	22	44	87	58	89	455

The number of patients who had an abnormal scan result was 184 (40%), whilst 239 (53%) had a normal scan result, and 32 (7%) had an equivocal scan.

### Pre-scan diagnosis outcomes

Suspected idiopathic PD accounted for 190 scans (42%), followed by suspected ET with 129 scans (29%) and DIP with 45 scans (10%). The total number of patients was 451. There were four scans (1%) where the pre-scan diagnosis was not able to be determined, due to insufficient information on clinical records, and thus, these were not included. For patients with a pre-scan diagnosis of PD, 100 patients (54%) had an abnormal scan, which was the largest proportion of patients. For patients with a pre-scan diagnosis of ET, 105 patients (44%) had a normal scan. Table 2 shows a further breakdown of the number and percentage of DaT scans for each pre-scan diagnosis and subsequent scan outcomes.

**Table 2:** The number and percentage of scans stratified by the pre-scan diagnosis from 2014 to 2021

	Number of abnormal scans	Abnormal scans (%)	Number of normal scans	Normal scans (%)	Number of equivocal scans	Equivocal scans (%)	Total number of scans (pre-scan diagnosis)
Possible PD	100	55.2	74	31.1	16	50.0	**190**
ET	16	8.8	105	44.1	8	25.0	**129**
LBD	18	9.9	8	3.4	2	6.3	**28**
DIP	11	6.1	31	13.0	3	9.4	**45**
Parkinson's plus	13	7.2	2	0.8	1	3.1	**16**
VP	13	7.2	11	4.6	2	6.3	**26**
Other diagnosis	10	5.5	7	2.9	0	0	**17**
Total number of scans (scan outcome)	**181**		**238**		**32**		
Grand total of scans							451

*PD* Parkinson’s disease, *ET* essential tremor, *LBD* Lewy-body dementia, *DIP* Drug-induced Parkinsonism, *VP* vascular parkinsonism

Bold values indicate the total number of scans (raw number)—It is calculated by the number of abnormal scnas+number of normal scans and number of equivocal scans in that row

Pre-scan diagnoses that were expected to have an abnormal DaT Scan result included PD, LBD, and Parkinson’s plus syndromes. Out of the total of 181 patients who had an abnormal scan, 131 patients (73%) had a pre-scan diagnosis that was consistent with the scan result. Out of the 238 patients with a normal scan, 154 patients (65%) had a pre-scan diagnoses that was consistent with this DaT result (including ET, DIP, VP, and other diagnoses).

### Post-scan diagnosis and clinical management

For all DaT scans, the diagnosis was changed for 168 patients (37%), whilst the clinical management was changed for 190 patients (42%).

The diagnosis was not changed in 283 patients (63%), whilst the clinical management was not changed in 260 patients (58%). There was insufficient information about the diagnosis and clinical management for four and five patients, respectively.

For all abnormal scans, 66 patients (37%) underwent a change in diagnosis, and 118 patients (65%) underwent a change in clinical management. For all normal scans, 91 patients (38%) underwent a change in diagnoses, whilst 51 patients (22%) underwent a change in management. For equivocal scans, 15 patients (36%) had a change in their diagnosis, whilst 13 patients (62%) underwent a change in management. Table 3 shows the number and percentage of people who had a change in diagnosis, as well as change in management.

**Table 3:** The number and percentage of people with a change in diagnosis and management stratified by the pre-scan diagnosis from 2014 to 2021

	Number of patients with a change in diagnosis	Patients who had a change in diagnosis (%)	Number of patients with a change in management	Patients who had a change in management (%)
Possible PD	98	55.7	87	45.3
ET	30	17.0	52	27.1
LBD	10	5.7	12	6.3
DIP	17	9.7	14	7.3
Parkinson's plus	5	2.8	9	4.7
VP	5	2.8	11	5.7
Other diagnosis	11	6.3	7	3.6
Total number of scans as per scan result	176		192	

*PD* Parkinson’s disease, *ET* essential tremor, *LBD* Lewy-body dementia, *DIP* Drug-induced Parkinsonism, *VP* vascular parkinsonism

**Fig. 1:**
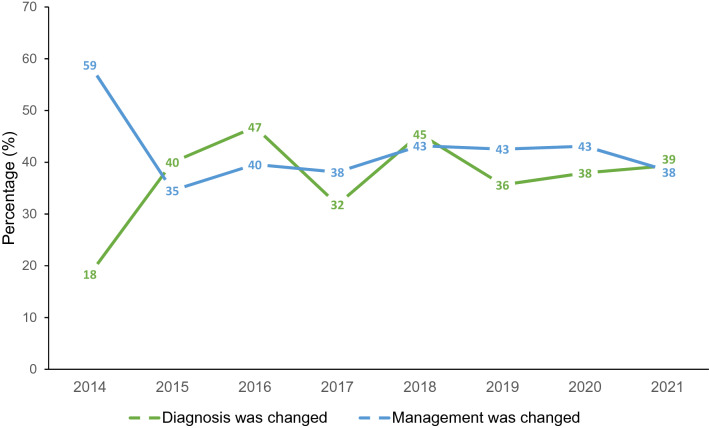
Percentage of change in diagnosis and clinical management from DaT scans from 2014 to 2021

For the patients who experienced a change in management, 119 patients (63%) had changes that involved starting dopaminergic medication. Dopaminergic medications were stopped for 10 patients (5%) out of those having a change in management, and 58 patients (31%) had other changes in management. This included starting medication for ET or LBD, stopping medication causing DIP, or referral to other specialists. Figure 1 shows the changes in trends of diagnosis and clinical management over the 8 years of the study.

Analysis of the duration between receiving the DaT scan and starting treatment was undertaken for the years 2018–2021, as these data were not available for the previous years. In these years, there were a total of 278 scans undertaken. The mean duration between the DaT Scan report and commencing treatment was 167 days, whilst the median duration was 91 days.

### Historical data

Although the complete dataset prior to 2014 was not available, there are historical data concerning the number of scans since 2004. These demonstrated that the number of scans has increased throughout the early years following the introduction of DaT scans, with an average of 9 scans per year in 2004–2005, 21 scans per year from 2006 to 2008 and 78 scans per year from 2010 to 2012. Historical data for 2009 and 2013 were not available, and so, the years provided are inclusive for all values.

In 2004–2005, 16 patients (89%) had scan results that confirmed the initial diagnosis, 16 scan results (89%) were abnormal, and 6 patients (33%) started treatment. In 2006–2008, 49 patients (78%) had scan results consistent with the expected diagnosis, 32 scan results (51%) were abnormal, and 52 patients (83%) had started treatment. In 2010–2012, 197 patients (84%) had scan results that confirmed the initial diagnosis, 101 scans (43%) were abnormal, and 134 patients (57%) subsequently initiated treatment.

**Fig. 2:**
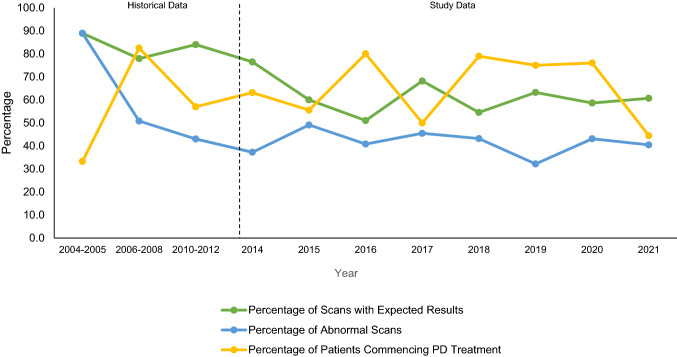
Trends in percentages for scan results that matched the expected diagnosis, percentages of abnormal scans, and percentages of patients starting PD treatment, for both historical and study data

Table 4 shows the number of scans per year, the percentage of scan results that confirmed the expected diagnosis, the percentage of abnormal scans, and the percentage of those starting treatment, for both historical and study data. Figure 2 demonstrates the trends graphically of the percentages for scan results that matched the expected diagnosis, percentages of abnormal scans and percentages of patients starting PD treatment, for both historical and study data.

**Table 4:** The number of scans per year, the percentage of scan results that matched the expected diagnosis, the percentage of positive scans, and the percentage of those starting treatment, for both historical and study data

Time period	Historical data			Study data							
	2004–2005	2006–2008	2010–2012	2014	2015	2016	2017	2018	2019	2020	2021
Number of scans	18	63	235	51	55	49	22	44	87	58	89
Scans per year (average number of scans for historical data)	9	21	78	51	55	49	22	44	87	58	89
Number of scans with expected results	16	49	197	39	33	25	15	24	55	34	54
Number of abnormal scans	16	32	101	19	27	20	10	19	28	25	36
Number of patients commencing PD treatment	6	52	134	12	15	16	5	15	21	19	16
Scans with expected results (%)	88.9	77.9	84.0	76.5	60.0	51.0	68.2	54.5	63.2	58.6	60.7
Abnormal scans (%)	88.9	50.8	43.0	37.3	49.1	40.8	45.5	43.2	32.2	43.1	40.4
Patients commencing PD treatment (%)	33.3	82.5	57.0	63.2	55.6	80.0	50.0	78.9	75.0	76.0	44.4

## Discussion

In this study, we aimed to determine the use of DaT scans in the diagnosis and clinical management of patients who exhibit features of Parkinsonism, where the cause is clinically not clearly distinguishable.

Over time, the average age at which patients were receiving DaT scans has increased. There was also a majority of males (57%) consistently found across all years. Males are more predisposed to developing PD and ET which is likely to have contributed to this finding [6, 7].

### Pre-scan diagnosis subgroups

Clinicians’ pre-scan diagnoses were generally consistent with the results of the DaT scans. For neurodegenerative Parkinsonism, pre-scan diagnoses were consistent with the scan 72% of the time. Conversely, for pre-scan diagnoses of non-neurodegenerative Parkinsonism, DaT scans were consistent 65% of the time.

There is some heterogeneity in the reported consistency between pre-scan diagnoses and DaT Scan result. A prospective study described poor correlation between pre-scan diagnoses and scan result, with a total of 36% of diagnoses and scan results not correlating for pre-synaptic Parkinson’s syndromes, and 54% not correlating for non-pre-synaptic Parkinson’s syndromes [8]. The study bears some similarity with our findings in that there is a higher accord between pre-scan diagnoses for neurodegenerative Parkinsonism scan results compared to non-neurodegenerative scan results, though there was a higher percentage of congruence between pre-scan diagnoses and scan results in this study. For non-neurodegenerative diagnoses, another study found the unanimity between pre-scan diagnosis and scan result was 65%, which is more comparable to our results [9].

### Post-scan diagnosis and management

For this study, there was a clinically significant change in the diagnosis and management from the use of DaT scans. A total of 37% of patients received a change in diagnosis, whilst 42% of patients experienced a change in management. These figures are consistent with the current existing literature [5, 10–12]. Previous studies have used the threshold of 30% to define a clinically significant change in outcome. A study using positron emission tomography to assess change in management for patients with dementia or cognitive impairment defined this threshold, and it was subsequently used by a systematic review that investigated the changes in management for patients undergoing DaT scans [5, 13]. The findings from this study therefore also support the use of DaT scans as being clinically significant in the diagnosis and management of these patients.

Out of the abnormal scans, 37% received a change in post-scan diagnosis, whilst 65% experienced a change in management, which meets the clinically significant threshold. However, for normal scans, whilst 38% of scans led to a change in diagnosis, only 22% resulted in a change in management. This is not surprising given that a negative scan would rule out neurodegenerative Parkinsonism and therefore may not warrant further dopaminergic treatment. Naturally, the final treatment decision would depend on the condition itself, the symptom severity, and the progression of the condition. Non-neurodegenerative movement disorders, such as VP and DIP, are often not treated with dopaminergic medications, and there is mixed evidence for their benefits [14–16]. As a result, using dopaminergic medication in non-neurodegenerative Parkinsonism is not usual in this service, especially given the potential harm of medication side-effects. For ET, patients will often choose to forgo further treatment if it does not affect their lives to a great degree [17].

For equivocal scans, 33% of scans resulted in a change in diagnosis, whereas 67% of scans resulted in a change in management. When requesting the scan, clinicians would often state the most-likely differential diagnosis based on the history and clinical examination. If the most-likely diagnosis was changed post-scan, then this was considered a change in diagnosis. From our data, there was a higher proportion of changes in management compared to changes in diagnosis. This may be attributed to clinicians choosing to treat empirically anyway despite ambiguity of the scan, especially if patient symptoms are severe or atypical. Patients who had equivocal scans generally did not have repeat scans, as they were followed-up clinically and for response to drug treatment if given. This often helped confirm diagnoses based on either progression or lack of progression of clinical findings. The results of the effects on diagnoses and management are therefore more resemblant to those with abnormal scans. It may be worthwhile to assess the outcomes of these patients in the long term in future studies.

Consistent with the current literature, there was a greater effect on change in management compared to change in diagnosis in all subgroups. This perhaps reflects the usage of DaT scans in clinical practice, where there are often used to confirm a suspected diagnosis rather than identify a different pathology [5].

### Duration to starting treatment

Between 2018 and 2021, the mean duration to the start of treatment was 167 days, whilst the median number of days was 91. This discrepancy and skew were likely due to outliers in the cohort who did not start treatment for several years, even after a positive DaT scan. In Parkinson’s disease, medication is generally given for symptomatic control and for improvement of quality of life, especially given that there is no evidence that current treatments slow the progression of the disease [18]. A randomized, non-inferiority trial demonstrated that providing treatment delayed by 40 weeks did not negatively affect overall Parkinsonian symptoms [19]. The study also found that there was no benefit or disadvantage to starting levodopa earlier compared to later, as there was previous contention about whether levodopa exerted a potential neurotoxic or neuroprotective effect [18–20]. The prolonged duration before initiating treatment in some patients in this study therefore reflects current clinical evidence, in that treatments are being started based on the clinical and symptomatic needs of the patient, rather than based purely on the result of the scan. These delays in starting treatment also demonstrate other factors in play when formulating the management plan.

That said, this is only relevant to the subset of our study where the outcome of the scan has resulted in the initiation of treatment. In patient with mild symptoms, DaT scans are still a valuable asset to rule out non-neurodegenerative disease, which would allow patients to access alternative treatment sooner. Alternatively, ruling out diagnoses of neurodegenerative Parkinsonism provides clinicians with more certainty when stopping unnecessary dopaminergic medication. The side-effects of medications for PD are well documented and include common side-effects such as nausea and vomiting and more serious side-effects, such as psychiatric symptoms [21]. Thus, avoiding unnecessary treatment of patients with dopaminergic agents is important.

From a broader perspective, negative DaT scan allows easier recognition of patients who would be appropriate for discharge from the Parkinson’s service. This ultimately helps reduce clinical pressures and renders the service more accessible to new patients awaiting assessment.

### Historical data

DaT scans were implemented in this service from 2004, from where there was an increase in the number of scans until 2010. From 2010, the number of scans has remained reasonably consistent. This is likely because the use of DaT scans had becoming more routine within the service. By 2010, there was increased experience relating to their utility.

The scans matched the expected diagnosis in the majority of cases, even in the early years of implementation, which is a trend that has continued in more recent years. This demonstrates the use of DaT scans as having a supportive confirmatory role in the overall diagnosis to the clinical diagnosis. There is more fluctuation regarding the percentages of those starting dopaminergic medication over time. However, this outcome is more variable, as patients are not started on dopaminergic medication solely based on the scan result. Despite this fluctuation, more than 30% of patients were commenced on dopaminergic medication in all years assessed, which therefore suggests that the use of DaT scanning has had a clinically significant effect from 2004 to 2021, as defined by the threshold previously mentioned.

With the exception of 2004–2005, the percentage of abnormal scans has also remained reasonably consistent, with figures ranging between 30 and 50% (see Fig. 2). It is speculated that 2004–2005 had a higher percentage of abnormal scans of 89% for two main reasons. The first is because there was only an average of nine scans per year in this time period, meaning that the value is more likely to be skewed due to the relatively few scans. The second is likely due to DaT scans being newly implemented within the movement disorder service at the time. Clinicians may have been more hesitant or felt that they needed a high threshold of suspicion of neurodegenerative Parkinsonism before considering scanning, given that the UK National Institute of Health and Care Excellence guidelines for DaT scanning only emerged in 2006 [22]. These guidelines would have likely provided clearer guidance on when DaT scanning should be used, which provides further explanation for the increase in DaT scans and why there is a greater consistency in the number of scan results that match the initial diagnosis from 2006 onwards.

### Strengths and limitations

This study has several strengths, as it includes one of the largest single study cohorts of patients for assessing the use of DaT scans in the diagnosis and management for patients with clinically indeterminate Parkinsonism. This study also assessed outcomes that were inadequately reported in the current literature, including the time between receiving the outcome of the DaT scan and commencing treatment. This was to provide more detailed insight on the clinical management in actual practice for patients.

The service is run by geriatricians with an interest in movement disorders but is not age-restricted. Previous PD prevalence research has demonstrated that it looks after the majority (more than 85%) of people with PD in the Northumbria catchment area [23, 24], which contributes to the strength of the study.

Some of the limitations of this study include the retrospective nature of the study, which meant that there was a proportion of patients, who were not included in one or more of the analyses due to the lack of information available. Although the number of these patients is relatively small, as described in the results, it is still worth noting.

The lack of initial documentation meant that for a few patients, pre-scan diagnosis could not be determined. For the post-scan diagnosis or clinical management, the main cause of missing information was due to loss to follow-up, and so their outcomes could not be ascertained. Both of these factors therefore contribute to attrition bias.

Additionally, this study only involved one centre, which limits the generalizability of the data. This study also did not assess the number of scans done by each clinician, and how this may change over time, due to increased experience. This may mean that there may be some variation between clinicians for the threshold for clinical uncertainty in DaT scan requests.

This study also did not correlate the clinicopathological data with post-mortem data, given that the majority of patients included were still living. However, this could be potentially an area of further research.

Finally, although DaT scans do contribute considerably to either changes or confirmation of diagnoses, it is unclear how much influence the results of DaT scans have on the overall change in management. Given that a management decision will likely be based on a variety of factors other than the scan result, including clinical severity of symptoms, quality of life, patient preference, and medication contraindications, the effect of using DaT scan on changing a management plan would be difficult to fully assess.

## Conclusion

This study concludes that DaT imaging is useful for supporting the correct diagnosis and clinical management for patients with clinically indeterminate Parkinsonism. There was a reasonable degree of consistency between clinicians’ pre-scan diagnosis and DaT scan results. However, management plans were not always initiated immediately after receiving the scan result, which was likely due to other factors, including disease severity and the disease impact. More research on assessing the effect of DaT scans on patient outcomes, including quality of life and symptom control, should therefore be undertaken.

## Data Availability

The participants of the study did not give written consent for their data to be shared publicly. Additionally, due to the confidential nature of the research data, the supporting data for this research is not available.
